# The effect of haemoglobin and blood transfusion on preterm infant gut perfusion and injury

**DOI:** 10.3389/fped.2024.1440537

**Published:** 2024-11-22

**Authors:** Claire Howarth, Christian Mifsud, Jayanta Banerjee, Simon Eaton, Terence Leung, Paul Fleming, Joan Morris, Narendra Aladangady

**Affiliations:** ^1^ Neonatal Unit, Homerton Healthcare NHS Foundation Trust and Queen Mary University of London, London, England; ^2^ Great Ormond Street Institute of Child Health, University College London (UCL), London, England; ^3^ School of Science and Technology, Centre for Health, Ageing and Understanding Disease (CHAUD), Nottingham Trent University, Nottingham, England; ^4^ Neonatal Unit, Imperial College Healthcare NHS Trust and Imperial College London, London, England; ^5^ Department of Medical Physics and Biomedical Engineering, University College London, London, England; ^6^ Population Health Research Institute, St George’s University of London, London, England

**Keywords:** haemoglobin, blood transfusion, preterm infants, gut perfusion, gut injury, NEC

## Abstract

**Introduction:**

There is significant uncertainty regarding the role that anaemia or red blood cell transfusion (RBCT) plays in the development of gut injury in preterm infants. This study evaluated Near Infrared Spectroscopy (NIRS) together with a range of known biomarkers of gut inflammation to identify their relationship with anaemia and RBCT.

**Method:**

A prospective observational study of preterm infants born at <30 weeks gestation was conducted from birth until either 36 weeks post conceptional age or discharge home. Gut perfusion and biomarkers of gut injury were assessed weekly by: 60 min NIRS measurements (splanchnic tissue oxygenation index [sTOI] and fractional tissue oxygenation extraction [sFTOE]); stool calprotectin; urine intestinal and liver fatty acid binding proteins (I-FABPs and L-FABPs); and trefoil factor 3 (TFF-3). Exclusion criteria included Fetal Growth Restriction (FGR), and abnormal antenatal Dopplers. Haemoglobin (Hb) levels were measured in parallel with NIRS measurements. NIRS, together with urine and stool biomarkers of gut injury, were evaluated up to 72 h pre/post RBCT and pre/post measurements compared.

**Results:**

Forty-eight infants were studied. Median (range) gestational age was 26 ^+^ ^3^ (23 ^+^ ^0^ to 29 ^+^ ^6^) weeks and birthweight 883.5 g (460–1,600). Seven (14.6%) infants developed ≥ Bells stage 2 NEC. 28 (58.3%), 5 (10.4%) and 24 (50%) infants had ECHO confirmed PDA, haemorrhagic parenchymal infarct (HPI) and IVH respectively. There were 22 episodes of sepsis. Infants were in the study for a median of 7.3 (1–13) weeks. There was no significant association between Hb divided into three categories (<80 g/L, 80–111.9 g/L and ≥120 g/L) or continuous values and sTOI, sFTOE or any of the gut injury biomarkers measured (*p* > 0.05). 283 RBCTs were administered; 117 (41.3%) within the first two weeks of life. Pre and post blood transfusion changes in splanchnic NIRS oxygenation, urine and stool gut injury biomarkers were measured in 165, 195 and 175 episodes of RBCT respectively. There was no significant post RBCT changes in splanchnic NIRS or gut injury biomarker levels (*p* > 0.05). However, post RBCT calprotectin was significantly reduced during the first 14 days of life (mean difference −114%, CI −185 to −42 & *p* 0.002).

**Conclusion:**

There was no association between anaemia or RBCT with NIRS measurements of tissue oxygen saturation and biomarkers of intestinal inflammation or gut injury in preterm infants enrolled in this study. Further studies with standardised methods of examining the relationship between anaemia, RBCT and gut injury are needed.

## Introduction

Necrotising enterocolitis (NEC) is one of the most common complications affecting preterm infants and carries significant morbidity and mortality (1). There remains great controversy regarding the association of anaemia and/or red blood cell transfusion (RBCT) with NEC development, despite numerous studies. Although incompletely understood (2), both polycythaemia and reperfusion injury are considered part of the pathogenesis of NEC (3).

There is evidence in the literature suggesting haemoglobin level may be related to gut inflammation (4) and several epidemiological studies report an association between RBCT and/or anemia, with a higher risk of NEC. There are also reports of a temporal association between RBCT and NEC with NEC typically occurring within 72 h of transfusion. Around 30% of reported NEC cases are temporally associated with RBCT and are sometimes referred to as “Transfusion Related NEC” (TR-NEC) or “Transfusion Related Acute Gut Injury”(TRAGI) (5–7). Conversely, other studies have shown no association between RBCT and NEC (8–12) and one retrospective cohort study showed that RBCT was actually protective against NEC (13). Furthermore, in recent animal (14–16) and human (17–19) studies, anaemia itself has been proposed to increase the risk of NEC, a concept supported by studies demonstrating that the use of erythropoietin (EPO) to treat anaemia may also decrease NEC (14, 20). Mohamed and Shah (21) performed a meta-analysis of observational studies on TR-NEC in 2012 and found increased odds of NEC within 48 h of receiving a RBCT. An updated meta-analysis in 2018 found no significant association between RBCT transfusion and NEC (8), but the quality of evidence remains low (22).

There is a wealth of literature examining biomarkers of gut injury; urinary and blood fatty acid binding proteins (FABPs) have been the most extensively studied (23–34), but trefoil factor 3 (TTF-3) (33–35) and stool calprotectin (36–41) have also been proposed as intestinal injury biomarkers in preterm infants. Near-Infrared Spectroscopy (NIRS) provides a non-invasive, contemporaneous bedside measurement of regional tissue oxygen saturation (rSO_2_) or tissue oxygenation index (TOI) reflecting perfusion and metabolism and could be a non-invasive marker of intestinal injury (42, 43). NIRS has been used in numerous studies to examine the relationship between RBCT and gut injury to try and resolve the debate as to whether RBCT causes gut injury and NEC (6, 44, 45).

The mechanisms underpinning why anaemia or RBCT could predispose an infant to NEC are unclear. Ischaemia, reperfusion, and inflammation are all known to affect intestinal blood flow and cause gut injury which might in turn underpin the pathophysiology of diseases like NEC. However, the association between anaemia, RBCT and gut injury is even less well described in the literature. In order to bridge the knowledge gap, this novel study examined how varying degrees of anaemia and RBCT influence measures of tissue oxygen saturation and biomarkers of intestinal inflammation or gut injury in preterm babies to identify whether there is any relationship between them.

## Materials and methods

### Study design and sample size

This was a prospective observational study conducted at a tertiary Neonatal Intensive Care Unit (NICU)—Homerton University Hospital (HUH), London. The neonatal unit (NU) at HUH is one of the largest in London providing more than 8,000 intensive and high dependency cot days annually. There are 16 ITU, 8 HDU and 22 SCBU cots. HUH is one of three regional perinatal centres in North Central and Northeast London and cares for amongst the highest proportion of babies born at 23- and 24-weeks gestation in England.

The population served by HUH has a high level of social deprivation; Hackney has significant levels of poverty and inequality ranking as the 18th most deprived local authority in England.^1^ The borough has high rates of overcrowding with a significant proportion of households living in social housing in addition to worse rates of income deprivation and higher rates of premature mortality compared with the rest of London boroughs.^2^ Furthermore around 40% of Hackney's population comes from Black and Minority Ethnic groups and it is well known that there are ethnic disparities in preterm infant morbidity and mortality. Compared to white infants, Black infants are twice as likely to be born preterm (46), and more likely to experience serious complications of prematurity including IVH, ROP, sepsis and NEC (47–51). Preterm infants with very low-birth-weight (VLBW), of which HUH admits a large proportion of infants each year, account for 70% of neonatal deaths and are predominantly Black ethnicity (52).

Other relevant unit practices at the time of this study: (1) maternal expressed breast milk (EBM) was used preferentially when enteral feeding was commenced and when not available, donor EBM (DEBM) was used for infants born at less than 28 weeks gestation or with a birthweight of less than 1,000 g; (2) probiotics were not used on the NU; (3) oxygen saturation targets were set at 90%–95% for preterm babies.

NIRS measurements and biomarkers of gut injury were measured weekly from birth until 36 weeks post conceptional age or discharge (to either home or their local hospital) from the NU. The study was approved by the Research Ethics Committee in the UK (REC reference: 16/LO/1353) and informed written parental consent was obtained.

At the time this study was designed limited data existed in preterm babies upon which to conduct robust power calculations. The closest study was an adult study examining the correlation between pre-transfusion I-FABP concentrations and changes in I-FABP concentrations following transfusion, which found a correlation of 0.37 in 50 adults who received a RBCT (53). Data from this adult study were used to calculate power, but the correlation might be expected to be stronger in infants, and perhaps up to 0.5. Therefore, to detect a 0.5 correlation between pre-transfusion I-FABP and changes in I-FABP after transfusions at the 5% level of significance and with 90% power, it was estimated that 43 infants were required.

### Objectives and hypothesis

We hypothesised that anaemia causes gut hypoperfusion and hypoxia which may trigger an inflammatory cascade causing gut tissue injury in preterm infants, and then subsequent blood transfusion induces a reperfusion injury of the gut in anaemic preterm infants.

Our primary objective was to investigate the association between haemoglobin level (Hb) and RBCT with intestinal perfusion and tissue injury. The primary outcome was a change in I-FABP in relation to anaemia and after RBCT. Secondary outcomes were changes in other urine and stool biomarkers of gut injury and NIRS readings measured in relation to anaemia and RBCT.

### Inclusion criteria

Eligible infants were those who were appropriately grown preterm infants born at less than 30 weeks gestation admitted to HUH NICU from October 2016 to May 2018. Infants were recruited by day seven after birth.

### Exclusion criteria

Infants with abnormal antenatal Dopplers, fetal growth restriction (defined as birthweight 2nd centile or less), major congenital anomalies and twin-to-twin transfusion syndrome were excluded. These groups of infants could have compromised gut perfusion meaning they are at an elevated risk of developing NEC, over and above their risk due to their prematurity.

### Measurements of gut perfusion

Weekly splanchnic tissue oxygenation index (sTOI) was measured for 60 min using a NIRS monitor (NIRO-300, Hamamatsu KK, Japan) along with concurrent measurement of peripheral arterial oxygen saturations using a pulse oximeter. For each 60-min recording, a mean sTOI was produced and then splanchnic fractional tissue oxygen extraction (sFTOE) was calculated using the equation [SaO_2_ – TOI]/SaO_2_) (54).

The NIRO-300 monitor is CE marked for clinical use in the neonatal population and uses reusable probes. The sensors were cleaned thoroughly between patients. The NIRS emitter and detector probes were placed on the infant's abdomen, just under the umbilicus for 60 min once a week by the same investigator (CH) to ensure uniform placement every time. The probes were placed in a protective rubber seal to ensure the distance between them was constant for each infant. The centre of this seal was lined up with the umbilicus meaning the actual emitter and detector probes were paramedian.

**Figure 1:**
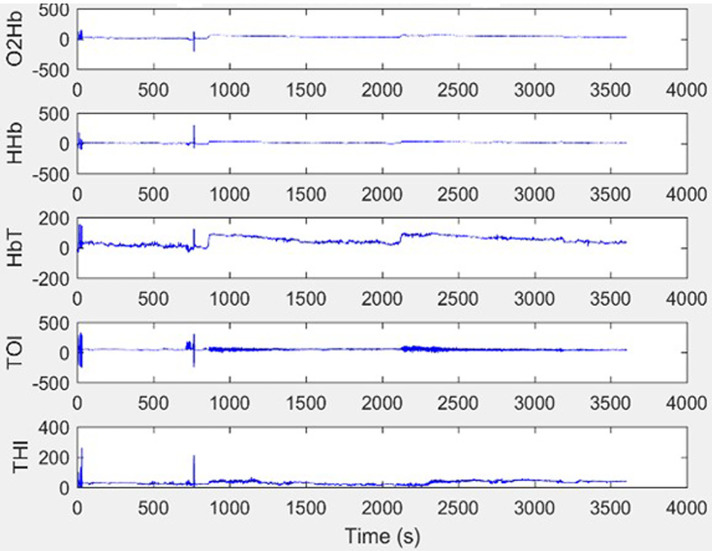
Example raw NIRS data without artefact. This show an excellent quality trace with minimal movement artefact.

CH was present during the entire measurement and if any movement artefact developed (seen on the NIRO monitor as a broadening of the raw data trace), it was addressed immediately. In the 3 infants where artefact was noticed, the cause was identified (in all cases this was due to the infant moving) and it was then ensured that the infants were swaddled appropriately within their bedding “nest” in their incubator to keep them comfortable and still, without interfering with the NIRS probes. In each of the 3 cases it took less than 5 min for the artefact to be noticed and resolved. All infants were on bolus feeds and the NIRS measurements were started at the onset of a feed for consistency. Using Matlab R2019b software (Mathworks, Natick, Massachussets, US) raw NIRS data were extracted for each infant for each week (Figure 1). Each NIRS reading was individually assessed for artefact and analysed in 5-minute epochs. Noisy epochs (representing artefact) were removed.

### Measurements of urine and stool biomarkers of gut injury

Weekly urinary intestinal fatty acid binding protein (I-FABP), liver fatty acid binding protein (L-FABP) and trefoil factor 3 (TTF-3) were measured. At least one ml of urine (by placing cotton wool in the nappy) was collected each week and placed in a universal container. As soon as each urine sample was collected, it was placed in a fridge on the NU which was set at four to five degrees Celsius. Samples were then transferred to the deep freezer for storage at −80 degrees Celsius. The maximum time that samples were left in the fridge before transfer to the freezer was 24 h, although most were moved within 6 h. Urine samples were transferred in a specialised container on dry ice to the Institute of Child Health (ICH), London for analysis.

Stool samples were collected weekly and placed in a fridge in the NU set at 4–5 degree Celsius. Stool samples were transferred to the same deep freezer for storage at −80C before transfer to ICH in the same period as for the urine samples.

### RBCT and haemoglobin (Hb) measurements

Hb level was measured weekly at the same time as the NIRS recording. Details and timing of any RBCTs an infant had received were recorded. There was no specific feeding policy around the time a RBCT was given, and in all infants their enteral feeds were continued as normal during a RBCT. TR-NEC typically occurs during or after the third week of life (5–7, 55) and 85 (30.1%) RBCTs were given from the third week onwards. We therefore examined the effect of RBCTs given in the first two weeks of life and those given over the entire study period. Pre transfusion NIRS readings were those measured within 72 h prior to RBCT and post transfusion NIRS readings were those measured within 72 h post RBCT. Infants who did not have any RBCT (*n* = 5) and those who did not have pre and post transfusion NIRS measurements performed (*n* = 7) were excluded from the analysis. For the gut biomarkers, pre transfusion gut tissue biomarkers in urine and stool were measured from samples obtained within 72 h prior to RBCT, and post transfusion gut tissue biomarkers in urine and stool were those measured from samples obtained within 72 h post RBCT. Infants who did not have any blood transfusions (*n* = 5) and those who did not have at least one pre and post transfusion marker measured were excluded from the analysis (*n* = 7).

### Additional data collected

Antenatal and perinatal maternal and infant characteristics were collected. Data were documented on volume of enteral feeds, level of cardiovascular/respiratory support, presence of PDA, sepsis (defined as culture positive or culture negative but meeting the European Medicines Agency definition (56), NEC (defined as ≥ Bells stage 2), Intraventricular Haemorrhage (IVH) and Haemorrhagic Parenchymal Infarct (HPI). Weekly blood results including maximum CRP and Haemoglobin (Hb) level at the time of the NIRS recording were collected.

### Statistical analysis

All statistical analyses were performed using STATA/SE version 15.1 (STATA Corp LLC, Texas, US). Multi-level mixed effects linear regression models were used. These were nested within each infant to allow for the fact that the readings were taken over time and hence were correlated within each infant. Confounding variables (presence of PDA, gender, volume of enteral feeding, haemoglobin, confirmed sepsis episode and gestational age) were included in the models as fixed effects.

To examine the effect of severity of anaemia, Hb level was divided into three groups; < 8 g/dl (group 1); 8–11.9 g/dl (group 2) and ≥12 g/dl (group 3). For the analysis of the effect of Hb level group 3 (i.e., a normal Hb) was taken as the baseline. The effect of haemoglobin level was also examined as a continuous variable. Differences between groups were analysed using Mann-Whitney U or Chi^2^ tests.

### Laboratory techniques for biomarker measurements

To measure urinary biomarkers a “sandwich” ELISA Kit was used. Each well of the ELISA plate is coated in the capture antibody specific to the target protein. To detect urinary I-FABP and L-FABP, ELISA kits from Hycult® Biotech were used and the manufacturer's instructions followed. To ensure accuracy when the plate was removed, each well was checked for bubbles which may have formed during the shaking process and if found, burst using a pipette tip and re-read. Standard curves were created for each plate by plotting the absorbance on the *y*-axis and log_10_ of the concentration on the *x*-axis. A four-parameter logistic regression model was used to tailor a line of best fit to the points and used to calculate the concentration of a given sample for both I-FABP and L-FABP.

TFF-3 was measured using RayBio® ELISA Kits and the manufacturer's instructions followed. As for FABP, if bubbles formed, the same method of removal was used as previously described. Standard curves were created for each plate by plotting absorbance on the *y*-axis and log_10_ of the concentration on the *x*-axis. A four-parameter logistic regression model was used to tailor a line of best fit to the points and used to calculate the concentration of a given sample.

The calprotectin kit from Hycult® Biotech was used to measure faecal calprotectin levels. To measure calprotectin accurately, samples were extracted using faecal extraction buffer. To breakup and homogenise the sample, a combination of vortexing and sonicating was used until no large particles remained, after which the samples were centrifuged for five minutes at 3,000 g. The supernatant was collected and stored at −80°C. A blank standard dilution buffer was also used alongside a high and low control both reconstituted with 0.25 ml of distilled water followed by the addition of 0.25 ml dilution buffer. These controls were used as a representation of what samples with high or low calprotectin concentrations would look like. Ideally any tested samples should lie between these two values with the correct dilution. The manufacturer's instructions detail running a test to determine the most suitable dilution to use for the samples, either 50 or 150×. To determine the suitable dilution factor, five samples were randomly chosen and run in duplicate alongside standards. When using 50× dilution, the standards ranged from 16 to 625 µg/g stool and from 48 to 875 µg/g stool when using a 150× dilution. The results of this test suggested that the 50× dilution was most suitable for our samples. Calprotectin standard curves were created by plotting the concentration of a given standard against its absorbance and a hyperbolic line of best fit. From this standard curve it was possible to determine the concentration of samples run alongside their given standards.

Creatinine was measured to standardise I-FABP, L-FABP and TFF-3 and account for changes in urine concentration between samples. This was completed by dividing the analyte concentration by the creatinine concentration. Urine creatinine was measured using a quantitative enzymatic (based on creatininase, creatinase, sarcosine oxidase and peroxidase) colorimetric assay kit (Sentinel Diagnostics). Standard curves were created by plotting the standard concentrations on the *x* axis and absorbance on the *y* axis. A line of best fit (linear regression) was used to calculate unknown sample concentrations for that plate.

## Results

### Infant characteristics

**Figure 2:**
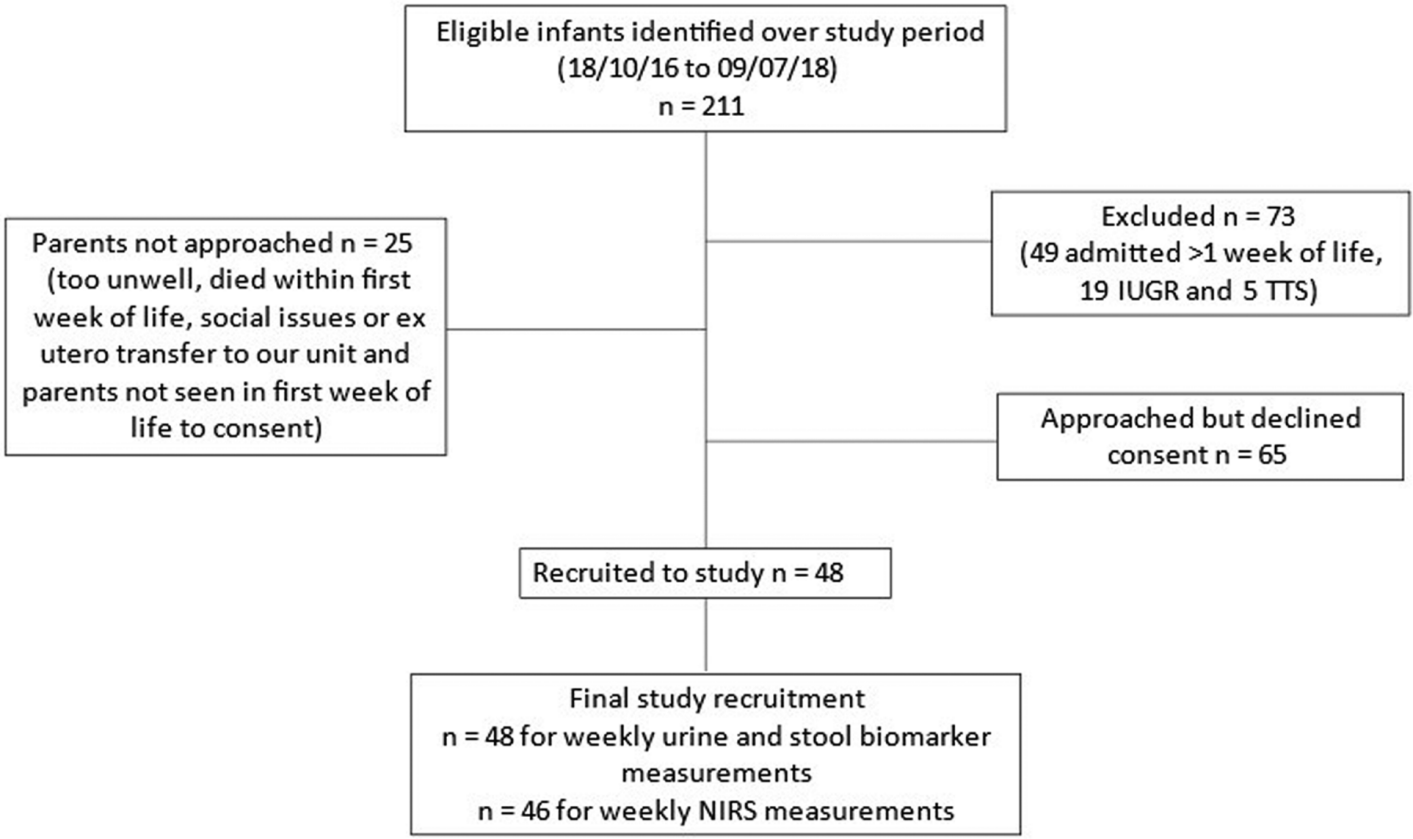
Study recruitment. TTS, twin to twin transfusion syndrome; IUGR, intra-uterine growth restriction.

There were 211 eligible infants admitted over the study period of which, forty-eight preterm infants born at <30 weeks GA were recruited. Details of numbers of eligible infants, exclusion and recruitment are presented in Figure 2. Median birth weight was 883.5 g (range 460 g–1,600 g), median gestation age was 26 ^+^ ^3^ weeks (range 23 ^+^ ^0^ to 29 ^+^ ^6^) and 52% were female. Parents of two infants withdrew their consent for NIRS measurements after one week but consented for urine and stool sample collection to continue. Therefore, 48 infants were eligible for inclusion in weekly urine and stool biomarkers of gut injury measurements and, 46 infants had the complete research measurements of weekly NIRS together with weekly urine and stool biomarkers of gut injury.

Demographic characteristics of the infants studied, those whose parents declined consent and those not approached (deemed too sick by the attending consultant's assessment or parents not available to consent during the infant's first week of life) are presented in Table 1. Two (4.2%) infants died (one from a tension pneumothorax in the first week of life and the other from NEC in the nineth week of life).

**Table 1:** Characteristics of the infants studied as well as those who declined consent and were not approached.

Characteristic	Infants studied (*n* = 48)	Approached but parents declined consent (*n* = 65)	Not approached (*n* = 25)
Birthweight (g)	883.5	900	855
[median (range)]	(460 to 1,600)	(425 to 1,750)	(569 to 1,400)
Gestational age (wks)	26 ^+^ ^3^	26 ^+^ ^6^	27 ^+^ ^3^
[median (range)]	(23 ^+^ ^0^ to 29 ^+^ ^6^)	(23 ^+^ ^0^ to 29 ^+^ ^4^)	(23 ^+^ ^3^ to 29 ^+^ ^6^)
Gender [number (%)]			
Male	23 (48.0)	32 (49.2)	13 (52.0)
Female	25 (52.0)	33 (50.8)	12 (48.0)
Ethnicity [number (%)]			
White (white British/white other)	30 (62.5)	40 (61.6)	15 (60.0)
Black (Afro Caribbean/black other)	10 (20.8)	14 (21.5)	6 (24.0)
Asian (Indian Asian/Pakistani/Asian other)	8 (16.7)	11 (16.9)	4 (16.0)

There were no significant differences between these groups for any listed characteristics (all *p* > 0.05).

### Additional maternal and infant characteristics

Antenatal maternal characteristics and infant characteristics are presented in Table 2.

**Table 2:** Antenatal maternal and postnatal infant characteristics.

Characteristic	Infants studied (*n* = 48)
Maternal chorioamnionitis [number (%)]	31 (64.6)
Antenatal steroids [number (%)]	
Complete course	30 (62.5)
Incomplete course	15 (31.3)
None	3 (6.2)
Days of ventilation [median (range)]	30 (1–65)
Days to achieve full feeds [median (range)]	21 (6–41)
Number of RBCT administered to each infant during NICU stay [median (range)]	5 (0–9)

### Infants diagnosed with NEC

Eight infants were treated for NEC with signs of abdominal distension, bilious aspirates and systemic features including metabolic acidosis, increased numbers of desaturations and bradycardias. Seven (14.6%) infants developed ≥ Bells stage 2 NEC. Only 1 out of the 7 infants required surgical treatment. There were no significant differences between infants with and without NEC, except for longer duration of ventilation in those infants with NEC [median (range) 26 days (6–48) compared with 6 days (1–65), *p* = 0.01]. NEC occurred between week 3 and 9 of life at corrected gestational ages ranging from 28 ^+^ ^6^ to 34 ^+^ ^3^ weeks.

### Sepsis episodes

All infants were started on prophylactic antibiotics at birth after blood cultures were taken. Of these, 6 infants had positive blood culture; 1 Coagulase Negative Staphylococcus, 1 Group B Streptococcus, 2 E. Coli, 1 Listeria and 1 Haemophilus Influenzae. There were 38 other instances of the infants being treated for sepsis but only two infants had positive blood culture. Fourteen of these infants met the EMA Sepsis definition (56). In total, there were 22 episodes of sepsis.

### Patent ductus arteriosus (PDA) and intracranial bleeds

PDA was confirmed on echocardiogram in 28 (58.3%) infants; 7 (14.6%) had no PDA. The remaining 13 (27.1%) did not have an echocardiogram. On cranial ultrasound scan, five infants (10.4%) had HPI and 24 (50%) had IVH. Of the 24 with ICH, 4 (16.7%) had Grade I IVH, 15 (62.5%) had grade II IVH and 5 (20.8%) had grade III IVH. There was no significant difference in grade of IVH or presence of PDA between those infants with NEC and those without (all *p* > 0.05).

### Length of time in the study

The median (range) number of weeks in the study was 7.3 (1–13) weeks for all 46 infants with NIRS measurements. Of these 46, 17 (35.4%) infants completed the study up to 36 weeks corrected gestational age and for the remaining 29 infants, the median (range) length of time in the study was 6 (1–8) weeks as they were either discharged home or back to their local hospital.

### NIRS measurements

In total 276 NIRS measurements (splanchnic Tissue Oxygenation Index [sTOI] and splanchnic Fractional Tissue Oxygenation Extraction [sFTOE]) were completed.

#### Association of NIRS measurements with Hb levels

Hb groups [severity of anaemia, <80 g/L (group 1); 80–119 g/L (group 2) and ≥120 g/L (group 3)] had no significant association with any of the NIRS measurements (Table 3). Haemoglobin level was also examined as a continuous variable, but no significant association was found with any of the NIRS measurements (all *p*-values > 0.05).

**Table 3:** Association of NIRS measurements with Hb levels.

NIRS measurements	Coefficient	Standard error	*P*-value	95% CI
sFTOE Hb group-1 vs. group-3	0.013	0.098	0.896	−0.170 to 0.205
sFTOE Hb group-2 vs. group-3	0.005	0.102	0.960	−0.195 to 0.205
sTOI Hb group-1 vs. group-3	−6.228	3.325	0.061	−12.745 to 0.288
sTOI Hb group-2 vs. group-3	4.925	7.777	0.527	−10.317 to 20.168

Haemoglobin (Hb): 3 groups- < 80 g/L (group 1); 80–111.9 g/L (group 2) and ≥120 g/L (group 3).

sTOI, splanchnic tissue oxygenation index; sFTOE, splanchnic fractional tissue oxygenation extraction.

#### Association of NIRS measurements with RBCT

A total of 283 RBCTs were administered; 81 (28.6%) in the first week after birth and 117 (41.3%) within the first two weeks. However, the pre and post transfusion NIRS splanchnic oxygenations were only measured in 165 RBCT episodes. There were no significant differences in pre and post transfusion splanchnic NIRS measurements of TOI and FTOE for blood transfusions given in the first 14 days after birth or over the entire study period (Table 4).

**Table 4:** Difference in NIRS readings pre and post RBCT.

NIRS reading	RBCT time period	Mean difference pre and post RBCT	95% CI	*p*-value
sTOI (%)	All Days	0.921	−3.071 to 4.912	0.651
sTOI (%)	<15 days	−2.910	−12.493 to 6.673	0.552
sFTOE	All Days	−0.005	−0.050 to 0.039	0.816
sFTOE	<15 days	0.003	−0.105 to 0.111	0.955

RBCT, red blood cell transfusion; sTOI, splanchnic tissue oxygenation index, sFTOE, splanchnic fractional tissue oxygenation extraction.

#### Confounding factors

Multiple linear regression analysis showed no significant association between confounding factors (gestational age, birthweight, gender, enteral feed volume, confirmed sepsis episode and presence of PDA) and NIRS measurements, (all *p*-values > 0.05).

### Gut biomarker measurements

#### Biomarker values

The values of I-FABP, L-FABP, TFF-3 and Calprotectin for infants enrolled into this study are presented in Table 5.

**Table 5:** Gut tissue biomarkers values for preterm infants <30 weeks gestation in the first 8 weeks of life^a^.

Week of life	Biomarker [values are presented in geometric mean (95% CI)]			
	I-FABP (pg/nmol creatinine)	L-FABP (pg/nmol creatinine)	TFF-3 (ng/nmol creatinine)	Calprotectin (ug/g stool)
1 (*n* = 48)	3.0 (2.1–4.3)	128.5 (73.6–224.3)	0.07 (0.06–0.09)	101.4 (57.2–179.6)
2 (*n* = 45)	3.4 (2.2–5.3)	78.8 (53.8–115.5)	0.06 (0.05–0.07)	96.5 (65.2–142.7)
3 (*n* = 37)	6.6 (4.5–9.8)	83.3 (54.3–127.6)	0.06 (0.04–0.07)	69.8 (45–108.4)
4 (*n* = 37)	9.0 (6.0–13.6)	108 (69.6–167.7)	0.07 (0.05–0.10)	70.6 (49.5–100.9)
5 (*n* = 33)	4.7 (2.6–8.5)	84.5 (49.1–145.5)	0.09 (0.06–0.13)	80.7 (55.6–117.2)
6 (*n* = 27)	6.9 (4.0–11.8)	89.2 (51.2–155.6)	0.08 (0.05–0.12)	65.9 (42.1–103.3)
7 (*n* = 22)	4.0 (2.1–7.5)	87.1 (51.3–147.9)	0.08 (0.05–0.13)	39.1 (20.9–73)
8 (*n* = 14)	8.7 (4.9–15.5)	155.4 (95.5–252.9)	0.09 (0.07–0.12)	49.0 (27.2–88.1)

^a^ Restricted to biomarkers measured during the first 8 weeks of life due to drop out in numbers each week as some infants were either discharged home or to their local hospital.

I-FABP, intestinal fatty acid binding protein; L-FABP, liver fatty acid binding protein; TFF-3, trefoil factor 3.

#### Association of biomarkers of gut injury with Hb

There was no association between any of the biomarkers measured and Hb level when analysed in the groups described above (Table 6). Haemoglobin level was also examined as a continuous variable but no significant association was found with any of the gut injury biomarkers measured (all *p*-values > 0.05).

**Table 6:** Association of gut biomarkers with haemoglobin levels.

Haemoglobin level	I-FABP Proportional difference (95%CI, *p*-value)	L-FABP Proportional difference (95%CI, *p*-value)	Calprotectin Proportional difference (95%CI, *p*-value)	TFF Proportional difference (95%CI, *p*-value)
Group-1 vs. Group-3	7% (−36% to +79%, *p* = 0.796)	−13% (−51% to 52%, *p* = 0.614)	−15% (−46% to 34%, *p* = 0.486)	−17% (−41% to 17%, *p* = 0.284)
Group-2 vs. Group-3	61% (−41% to +337%, *p* = 0.351	10% (−61% to 206%, *p* = 0.86)	45% (−53% to 352%, *p* = 0.517)	64% (−17% to 222%, *p* = 0.153)

Haemoglobin: 3 groups- <8 g/dl (group 1); 8–11.9 g/dl (group 2) and ≥12 g/dl (group 3).

I-FABP, intestinal fatty acid binding protein; L-FABP, liver fatty acid binding protein; TFF-3, trefoil factor 3.

#### Association of biomarkers of gut injury with RBCT

One hundred and ninety five RBCT episodes were examined for urine biomarkers of intestinal injury (I-FABP, L-FABP and TFF-3) and 175 RBCT episodes for stool calprotectin. There were no significant differences in pre and post RBCT I-FABP, L-FABP, TFF-3 or calprotectin measurements when examined for RBCT given over the entire study period. However, when these were examined for RBCTs given only in the first 14 days after birth, a significant decrease in calprotectin levels post RBCT was seen (Table 7).

**Table 7:** Difference in biomarker readings pre and post RBCT.

Markers	Transfusions	Percentage difference (as log scale^a^)			
		Mean difference	Lower 95% CI	Upper 95% CI	*p*-value
I- FABP	All Days	10%	−14%	34%	0.434
I- FABP	<15 days	50%	−2%	102%	0.061
L- FABP	All Days	−8%	−30%	15%	0.503
L- FABP	<15 days	−12%	−62%	39%	0.652
TFF-3	All Days	1%	-18%	20%	0.926
TFF-3	<15 days	6%	−36%	47%	0.792
Calprotectin	All Days	−21%	−51%	9%	0.169
Calprotectin	<15 days	−114%	−185%	−42%	**0**.**002**

The bold value indicates the post RBCT calprotectin which was significantly reduced during the first 14 days of life (0.002).

^a^ Converted to log scale as the biomarkers all showed a non-Gaussian distribution.

#### Confounding factors

Multiple linear regression analysis showed no significant association between confounding factors (gestational age, birthweight, gender, enteral feed volume, confirmed sepsis episode and presence of PDA) and gut biomarker measurements, (all *p*-values > 0.05).

## Discussion

This novel study examined the effect of both Hb level and RBCT on the combination of splanchnic oxygenation, using NIRS measurements and multiple biomarkers of gut tissue injury. No association between anaemia or RBCT with either NIRS measurements of tissue oxygen saturation, or urine and stool biomarkers of gut injury, was demonstrated in preterm infants enrolled in this study.

### Effect of Hb and RCBT on gut oxygenation

Gut perfusion, examined by 60-minute NIRS recordings, demonstrated no effect of Hb level or RBCT on splanchnic regional oxygenation in this study. In this cohort, neither anaemia nor RBCT caused gut injury at the tissue level and represented by changes in NIRS measurements of gut oxygenation.

The direct impact of anaemia on splanchnic oxygenation as measured by NIRS has not been as extensively studied in preterm infants as the effect of RBCT, but similar to our study, Bailey et al (57) found no correlation between cerebral or splanchnic oxygenation and Hb level. In our study, we chose to use 72 h pre and post RBCT as the period in which to examine the impact of RBCT on NIRS measurements. This is because most of published data on TR-NEC suggest that this condition develops within 48–72 h of a transfusion being given (5–7, 58, 59). The impact of RBCT on regional tissue oxygenation measured by NIRS has been studied in both adults and infants but demonstrates variable results depending on the region of the body studied. Studies looking at cerebral oxygenation show improvements in cTOI after RBCT which persist after 24 h (60). However, these studies are small and look at changes in cerebral oxygenation only (and not splanchnic oxygenation). Other studies demonstrate that an improvement in regional oxygenation after RBCT only occurs for up to 24 h post RBCT: Bailey et al (57) examined regional cerebral and splanchnic oxygenation in relation to RBCT in 30 infants but only recorded this for 20 min immediately before, during, immediately after, and 12 h after RBCT. They reported significantly increased regional oxygenation at 12 h (only) after the RBCT. Dani et al (61) used NIRS monitoring during RBCT in 15 preterm infants with symptomatic anaemia of prematurity (haematocrit level of <25%) from 60 min before the beginning of RBCT to 60 min after the RBCT, and confirmed that RBCT caused an increase in cerebral, splanchnic, and renal regional oxygenation. Banerjee et al. also examined this and found increased splanchnic oxygenation up to 60 min post RBCT (62, 63).

However, all these studies examined the effects of RBCT on regional oxygenation for up to 24 h after RBCT and demonstrate persistence of increased tissue oxygenation following RBCT at 24 h. Our study differs as the length of time we evaluated for is greater (up to 72 h) and our findings suggest that the increased oxygenation demonstrated in the aforementioned studies is not sustained beyond 24 h.

There is also an inherent problem with comparing levels of splanchnic oxygenation using NIRS between studies as the position of the probes varies. Furthermore, measurements can be affected by faecal content and abdominal gas (leading to distension). Abdominal gas is a particularly pertinent issue in preterm babies due to the use of non-invasive respiratory support as part of routine neonatal care (64) and could contribute to substantial variation in results across studies.

We must also consider the impact of the regulation of blood flow in response to hypoxia where brain-sparing occurs in preference to the gut and peripheral tissue perfusion. Balegar et al (65) conducted a prospective cohort study to evaluate if this hierarchical sequence alters in anaemic preterm infants (*n* = 30, median birthweight 923 g and gestational age 26.4 weeks) after a RBCT. The authors compared 4 h mean pre-transfusion cerebral and splanchnic FTOE with hourly means of cerebral and splanchnic FTOE during transfusion (4 h) and 24 h after RBCT. They reported that cerebral FTOE significantly decreased during and for 24 h after RBCT demonstrating improved cerebral oxygenation but found no significant changes in splanchnic FTOE. The authors concluded that cerebral perfusion follows a hierarchical response in anaemic infants after a RBCT. However, splanchnic perfusion did not improve following a RBCT as noticed in our study. Balegar and colleagues hypothesised that this lack of improvement in splanchnic perfusion following a RBCT in anaemic preterm infants may indicate continued splanchnic hypoxia that could predispose to TA-NEC.

Aktas et al (66) examined the impact of both severity of anaemia pre transfusion and this hypothesis of brain-sparing physiology affecting NIRS measurements. They reported that cerebral oxygenation did not change significantly, but that abdominal regional oxygenation increased at 24 h after RBCT in anaemic infants. The authors concluded that the increase in splanchnic oxygenation after RBCT might suggest reduced tissue oxygenation of the intestines during severe anaemia, but that the lesser impact of RBCT on cerebral oxygenation after transfusion might be explained by a brain-sparing mechanism.

It is important to note that direct comparisons of outcomes in these studies are challenging. Each uses different transfusion practices, different age of red blood cells when transfused, different time intervals between RBCT and NIRS measurement and all are conducted across different time periods with different populations.

### Effect of anaemia and HB level on urine and stool biomarkers of gut injury

No association of Hb levels with any of the biomarkers evaluated in this study was found. However, this may have occurred because the samples were collected for up to 72 h after Hb measurement. This time period may be too long to capture any effect of anaemia on biomarker levels. When intestinal mucosal damage occurs causing the integrity of the gut membrane to be impaired, FABPs are rapidly released into the circulation and their plasma concentration and subsequent urine concentration increases (25, 28, 31, 67, 68). Relative hypoxia occurs when anaemia is present. Animal models of anaemia demonstrate the activation of hypoxia-inducible factor (HIF) in response to anaemia (69). This subsequently causes transcriptional upregulation of many genes that improve the intestinal epithelial cell barrier function and in turn improve gut membrane integrity. In the present study, urine and stool samples used to measure gut injury biomarkers were collected for up to 72 h after Hb measurement. It is plausible, that the 72 h window between Hb and gut biomarker measurements, was long enough for these epithelial cell barrier reparative transcription changes to occur, which might explain the findings in our study.

NEC is a multifactorial disease and inflammation is known to contribute to its pathophysiology. Researchers have previously demonstrated elevated levels of I-FABP or L-FABP in cases of NEC (67, 70). Anaemia has also been shown to increase the risk of NEC (17) and one of the mechanisms by which this may occur is through the production of IFN-gamma. A recent animal study (71) demonstrated that increased levels of IFN-gamma correlated with increasing severity of anaemia, (phlebotomy induced). IFN-gamma is a potent pro-inflammatory cytokine implicated in intestinal inflammation and injury and its production may predispose an infant to NEC (71).

### Effect of RBCT on urine and stool biomarkers of gut injury

No effect of RBCT was found on any of the urine and stool biomarkers of gut injury (collected for up to 72 h after a RBCT) in this study. This 72 h time frame was deemed to be appropriate as previous studies have shown that I-FABP is raised for up to 48 h after initial gut injury (72), and TR-NEC is shown to develop within 48–72 h of a RBCT (5–7). In clinical practice, measuring the effect of RBCT on urine and stool samples in babies is always challenging as their passage is highly variable and unpredictable.

Researchers looking at TR-NEC hypothesise that RBCTs induce a pro-inflammatory response, leading to NEC. Dani *et al* (73) prospectively examined 20 infants less than 32 weeks gestation, and showed significant increases in IL1- Beta, IL-8, IFN gamma, IL-17, MCP-1, IP-10, and ICAM-1 after RBCT. However, levels of IL-6 and TNF-alpha, two cytokines known to play a key role in NEC development, were unchanged. Whilst rises in pro-inflammatory have been documented after RBCT, it is not known whether these rises correlate with inflammation and gut tissue injury. Our study suggests this may not be the case, but further confirmatory studies are needed.

Other factors that need to be considered and which may differ between studies include the processing and storage of red blood cells (RBCs) and the age of RBC at the time they are transfused. Age can reduce the deformability of RBCs and increase their adhesion aggregation. In turn, this increases the risk of blockage in the microcirculation of the splanchnic bed (74). RBCs used for transfusion in this study were aged up to the end of day 35 as per national UK guidance (75).

In our study there was a significant decrease in calprotectin levels post RBCT in the first 14 days after birth. This would challenge the hypothesis that RBCTs induce a pro-inflammatory response. Ho *et al* (4) measured faecal calprotectin pre and post RBCT in VLBW infants and showed that calprotectin levels increased after RBCTs. However, calprotectin levels are also influenced by the haematocrit level, the length of time the RBC had been in storage as well as other factors including: antibiotic use; volume of enteral feeds; and gastrointestinal colonisation (76). Many of these factors will be different between our study and that of Ho et al. Other factors that might account for differences between the present study and Ho's include the impact of postnatal age and any differences in the blood products used. Xu and colleagues have shown that calprotectin naturally decreases during the first two weeks after birth in preterm infants (77). Furthermore, the first 14 days are a time when the transitional period from fetal to extra-uterine life is most active, and when intensive care is at its highest in terms of cardiorespiratory support and the number of interventions an infant requires. All of these factors may have impacted our results.

Published data from the ETTNO (78) and TOP (79) trials have enhanced our knowledge of the effect of RBCTs on rates of NEC when exploring different RBCT transfusion thresholds. The ETTNO trial examined the effects of liberal vs. restrictive transfusion thresholds among 1,013 infants with birth weights of 400 g to 999 g in six level III/IV NICUs in Europe. Infants were randomly assigned to liberal (*n* = 492) or restrictive (*n* = 521) RBCT thresholds based on their postnatal age and health status. The TOP trial was a multicentre trial in which infants with a birth weight of 1,000 g or less, and a gestational age between 22 weeks and 28 weeks plus 6 days, were randomly assigned within 48 h after delivery to receive RBCT at higher or lower haemoglobin thresholds until 36 weeks of postmenstrual age or discharge. Although these studies were underpowered to examine the effect of the primary outcome on NEC, neither trial reported a significant difference in NEC rates between the two transfusion threshold groups. Data from these trials adds weight to our study supporting evidence justifying the adoption of lower hemoglobin thresholds for RBCT in preterm infants. Alternatively, it could reflect that regular RBCTs (given in the higher threshold groups) prevent anaemia and thus prevent ischaemia reperfusion mechanisms from taking place. Recently, Salas et al (12) published their secondary analysis from patients enrolled in the TOP trial. This analysis looked specifically at whether there is a temporal association between 72-h periods of exposure to RBCT, and NEC. In their *post hoc* analysis, the authors reported that exposure to RBCT was not associated with a higher risk of NEC.

### Strengths

There are several strengths of our study including: the number of measurements completed for both NIRS and gut biomarkers; the minimal impact of motion artefact on NIRS measurements; and the statistical analyses. Confounding variables (presence of PDA, gender, volume of enteral feeding, haemoglobin, and gestational age) were included in the models as fixed effects. This differs to previous studies where these confounding variables have not always been accounted for (80, 81).

### Population studied

Infants born at less than 30 weeks gestation represent those at highest risk of developing intestinal pathology including NEC and in contrast with previous studies where NIRS splanchnic oxygenation studies have focused on more mature infants, this study included more extreme preterm infants (80, 81). NEC among infants enrolled in this study occurred in 14.8% of participants. All cases occurred in babies born at less than 28 weeks. NEC has previously been reported to occur in 14% infants <26 weeks gestation and 10% < 31 weeks gestation (82). Although the NEC rate in our cohort was higher than the national average, it remains comparable to other centres in inner city South East England (82).

### NIRS measurements

The present study completed 276 splanchnic NIRS oxygenation measurements. This represents one of the largest numbers of NIRS measurements performed in a single cohort in published literature including studies examining cerebral oxygenation, which is more frequently reported (80, 81, 83–85). Previous studies examining splanchnic oxygenation often focus on early oxygenation measurements after birth and during the acute transitional period, and/or over the first postnatal month (80, 81). Our study also demonstrates the feasibility of using splanchnic NIRS in the most immature neonatal populations.

In most NIRS studies around 10%–15% of infants are excluded due to motion artefact (62, 86). No participants were excluded in this study because the investigator (CH) was present for the entire recording and was able to address any motion artefact immediately. Furthermore, inter-operator variability was eliminated as all NIRS probe placements were maintained by the same investigator (CH). NIRS analyses were completed in 5-min epochs and any areas within the 60-min recording deemed “noisy” (representing artefact) were removed from the analysis under the guidance of a medical physicist. In this study, only three recordings in three different infants had epochs removed, accounting for only one epoch during each recording.

### Urine and stool biomarkers of gut injury measurements

Three hundred and thirty two urine and 324 stool samples were collected. This represents a comparably large data set for such biomarkers in a preterm patient population. Previous studies have measured the ratio between I-FABP levels in urine (I-FABPu) and urinary creatinine to compensate for variation in urine concentration (41, 69–71). In this study, biomarker measurements were standardised and corrected for urinary creatinine as urine output in preterm infants is often variable.

### Limitations

The main limitations of the study included: the power calculation; timing of urine and stool samples collected in relation to the RBCT; and duration of NIRS measurements.

### Power calculation

The sample size estimate was based on an adult study of 50 episodes of RBCT and I-FABP changes (53). No similar study existed in preterm newborn infants at the time this study was designed. The estimated sample size was 43 infants but, paired pre and post RBCT gut injury biomarkers were measured in only 36 infants. However, paired pre and post transfusion NIRS, urine and stool biomarkers were measured in 165, 195 and 175 RBCT episodes respectively. Furthermore, the narrow confidence interval (CI) observed between RBCT, and gut injury biomarker results suggests that achieving the estimated sample size target would not have materially changed the results.

### Timing of sample collection and biomarker measurements

The estimated difference in biomarkers around RBCT and anaemia could be imprecise. The Hb values were divided into three groups (<80 g/L, 80–111.9 g/L and ≥120 g/L) to examine the effect of severity of anaemia on gut. This resulted in a smaller sample size in each of the three Hb groups, however, Hb was also analysed as a continuous variable and confirmed the absence of significant association with gut injury biomarkers. Temporality between exposure (anemia and RBCT) and changes in the biomarkers measured is difficult to determine.

Collecting urine and stool samples from extreme preterm infants is unpredictable and difficult. A time window of up to 72 h pre and post RBCT was used because of uncertainties that infants would pass stool and urine. The exposure and the time window of up to 72 h for urine and stool sample collection may be too long to detect differences in gut injury biomarkers measured in this study (33). Also, it is plausible that the effect of severity of anaemia or RBCT may not have been detected due to wide variation in urine (I-FABP, L-FABP and TFF-3) and stool (Calprotectin) biomarker values documented in this study.

Splanchnic NIRS biomarkers were only measured once a week for 60 min and may have been insufficient to identify the actual changes in gut perfusion related to anaemia and RBCT. Continuous NIRS measurements pre, during and up to 48–72 h post RBCT would have given more information on gut perfusion changes and on the duration such changes persist. However, the time gap between the exposure (RBCT/anaemia) and measurement of gut injury markers in the present study is reasonable as I-FABP is raised for 48 h after initial gut injury (72), and TR-NEC has been shown to develop within 48–72 h of a RBCT (5–7). Future studies using continuous NIRS monitoring are likely to provide more detailed insights.

## Conclusion

This study showed no association between anaemia or RBCT with NIRS measurements of tissue oxygen saturation and gut biomarkers of intestinal inflammation or gut injury, in a cohort of preterm infants. Our findings support the argument that the pathophysiology of NEC is complex and that anaemia and/or RBCT alone, do not cause gut injury, *or* that they do not cause gut injury detectable by NIRS or by measurement of common urine and stool biomarkers of gut injury evaluated in this study.

However, because many authors have suggested that anaemia and/or RBCT can lead to the development of NEC this in turn has led to differences in blood transfusion recommendations amongst clinicians. Our findings could potentially translate into changes in the clinical care of preterm babies. As an example, we provide evidence at tissue level justifying recent recommendations to adopt lower Haemoglobin thresholds for RBCT for preterm infants (87), which in turn, may reduce the risks associated with multiple blood transfusion.

Amongst all studies examining the relationship between anaemia, RBCT and NEC, there are differing clinical practices regarding when to transfuse, differing feeding policies during RBCT, and differing time intervals between RBCT and any measurements of gut injury or oxygenation. Standardising these factors in future studies is essential. Furthermore, the inclusion of preterm infants known to be at higher risk of developing NEC [e.g., fetal growth restricted (FGR) preterm infants] will be invaluable.

## Data Availability

Existing datasets are available in a publicly accessible repository: Publicly available datasets were analyzed in this study. This data can be found here: https://doi.org/10.24376/rd.sgul.22928984.v1.

## Footnotes

^1^ https://cityhackneyhealth.org.uk/population-infographics/

^2^ https://trustforlondon.org.uk/data/boroughs/hackney-poverty-and-inequality-indicators/

## References

[1] Han SM Hong CR Knell J Edwards EM Morrow KA Soll RF Trends in incidence and outcomes of necrotizing enterocolitis over the last 12 years: a multicenter cohort analysis. J Pediatr Surg. (2020) 55(6):998–1001. 10.1016/j.jpedsurg.2020.02.046 32173122

[2] Howarth C Banerjee J Aladangady N. Red blood cell transfusion in preterm infants: current evidence and controversies. Neonatology. (2018) 114:7–16. 10.1159/000486584 29550819

[3] Lucas A Cole TJ. Breast milk and neonatal necrotizing enterocolitis. Lancet. (1990) 336(8730):1519–23. 10.1016/0140-6736(90)93304-8 1979363

[4] Ho TT Groer MW Luciano AA Schwartz A Ji M Miladinovic BS Red blood cell transfusions increase fecal calprotectin levels in premature infants. J Perinatol. (2015) 35(10):837–41. 10.1038/jp.2015.73 26181719 PMC6368852

[5] Mally P Golombek SG Mishra R Nigam S Mohandas K Depalhma H Association of necrotizing enterocolitis with elective packed red blood cell transfusions in stable, growing, premature neonates. Am J Perinatol. (2006) 23(8):451–8. 10.1055/s-2006-951300 17009195

[6] Marin T Moore J Kosmetatos N Roback J Weiss P Higgins M Red blood cell transfusion –related necrotising enterocolitis in very low birthweight infants: a near infrared spectroscopy investigation. Transfusion. (2013) 53:2650–8. 10.1111/trf.12158 23480548 PMC3686850

[7] Josephson CD Wesolowski A Bao G Sola-Visner MC Dudell G Castillejo MI Do red cell transfusions increase the risk of necrotizing enterocolitis in premature infants? J Pediatr. (2010) 157(6):972–8. 10.1016/j.jpeds.2010.05.054 20650470 PMC4425198

[8] Garg P Pinotti R Lal CV Salas AA. Transfusion-associated necrotizing enterocolitis in preterm infants: an updated meta-analysis of observational data. J Perinat Med. (2018) 46(6):677–85. 10.1515/jpm-2017-0162 29176013

[9] Sharma R Kraemer DF Torrazza RM Mai V Neu J Shuster JJ Packed red blood cell transfusion is not associated with increased risk of necrotizing enterocolitis in premature infants. J Perinatol. (2014) 34(11):858–62. 10.1038/jp.2014.59 25144159 PMC4584142

[10] Wallenstein MB Arain YH Birnie KL Andrews J Palma JP Benitz WE Red blood cell transfusion is not associated with necrotizing enterocolitis: a review of consecutive transfusions in a tertiary neonatal intensive care unit. J Pediatr. (2014) 165(4):678–82. 10.1016/j.jpeds.2014.06.012 25039042 PMC4845907

[11] Harsono M Talati A Dhanireddy R Elabiad M. Are packed red blood cell transfusions protective against late onset necrotizing enterocolitis in very low birth weight infants. E-PAS. (2011) 2011:509.

[12] Salas AA Gunn E Carlo WA Bell EF Das A Josephson CD Timing of red blood cell transfusions and occurrence of necrotizing enterocolitis: a secondary analysis of a randomized clinical trial. JAMA network Oopen. (2024) 7(5):e249643. 10.1001/jamanetworkopen.2024.9643 PMC11069076 38700862

[13] Sood BG Rambhatla A Thomas R Chen X. Decreased hazard of necrotizing enterocolitis in preterm neonates receiving red cell transfusions. J Matern Fetal Neonatal Med. (2016) 29(5):737–44. 10.3109/14767058.2015.1016422 25731658

[14] Guneli E Cavdar Z Islekel H Sarioglu S Erbayraktar S Kiray M Erythropoietin protects the intestine against ischemia/reperfusion injry in rats. Mol Med. (2007) 13(9-10):509–17. 10.2119/2007-00032.Guneli 17873970 PMC1976860

[15] Shiou SR Yu Y Chen S Ciancio MJ Petrof EO Sun J Erythropoietin protects intestinal epithelial barrier function and lowers the incidence of experimental necrotizing enterocoloitis. J Biol Chem. (2011) 8(286(14)):12123–32. 10.1074/jbc.M110.154625 PMC3069416 21262973

[16] Kumral A Baskin H Duman N Yilmaz O Tatli M Ozer E Erythropoietin protects against necrotizing enterocolitis of newborn rats by the inhibiting nitric oxide formation. Biol Neonate. (2003) 84(4):325–9. 10.1159/000073642 14593244

[17] Patel RM Knezevic A Shenvi N Hinkes M Keene S Roback JD Association of red blood cell transfusion, anemia, and necrotizing enterocolitis in very low-birth-weight infants. JAMA. (2016) 315(9):889–97. 10.1001/jama.2016.1204 26934258 PMC4805423

[18] Ledbetter DJ Juul SE. Erythropoietin and the incidence of necrotizing enterocolitis in infants with vey low birth weight. J Pediatr Surg. (2000) 35(2):178–81. 10.1016/S0022-3468(00)90006-X 10693662

[19] Qi W Shen Q Zhang L Han LP Wang S. Study on the inflammatory intervention of erythropoietin on nec. Exp Ther Med. (2016) 11(6):2221–4. 10.3892/etm.2016.3199 27284304 PMC4887799

[20] Wang Y Song J Sun H Xu F Li K Nie C Erythropoietin prevents necrotizing enterocolitis in very preterm infants: a randomized controlled trial. Transl Med. (2020) 18:308. 10.1186/s12967-020-02459-w PMC7414749 32771013

[21] Mohamed A Shah PS. Transfusion associated necrotizing enterocolitis: a meta-analysis of observational data. Pediatrics. (2012) 129(3):529–40. 10.1542/peds.2011-2872 22351894

[22] Hay S Zupancic JA Flannery DD Kirpalani H Dukhovny D. Should we believe in transfusion-associated enterocolitis? Applying a grade to the literature. Semin Perinatol. (2017) 41(1):80–91. 10.1053/j.semperi.2016.09.021 27866662

[23] Coufal S Kokesova A Tlaskalova-Hogenova H Snajdauf J Rygl M Kverka M. Urinary intestinal fatty acid-binding protein can distinguish necrotizing enterocolitis from sepsis in early stage of the disease. J Immunol Res. (2016) 216:1–8. 10.1155/2016/5727312 PMC4823515 27110575

[24] Derikx JP Evenett NJ Degraeuwe PL Mulder TL van Bijnen AA van Heurn LWE Urine based detection of intestinal mucosal damage in neonates with suspected necrotising enterocolitis. Gut. (2007) 56:1473–5. 10.1136/gut.2007.128934 17872576 PMC2000285

[25] Edelson MB Sonnino RE Bagwell CE Lieberman JM Marks WH Rozycki HJ. Plasma intestinal fatty acid binding protein in neonates with necrotizing enterocolitis: a pilot study. J Pediatr Surg. (1999) 34(10):1453–7. 10.1016/S0022-3468(99)90102-1 10549746

[26] Gollin G Marks C Marks WH. Intestinal fatty acid binding protein in serum and urine reflects early ischemic injury to the small bowel. Surgery. (1993) 113(5):545–51. 8488474

[27] Gollin G Stadie D Mayhew J Slater L Asmerom Y Boskovic D Early detection of impending necrotizing enterocolitis with urinary intestinal fatty acid-binding protein. Neonatology. (2014) 106(3):195–200. 10.1159/000362497 25012466

[28] Guthmann F Borchers T Wolfrum C Wustrack T Bartholomaus S Spener F. Plasma concentration of intestinal- and liver-fabp in neonates suffering from necrotizing enterocolitis and in healthy preterm neonates. Mol Cell Biochem. (2002) 239(1-2):227–34. 10.1023/A:1020508420058 12479590

[29] Heida FH Hulscher JBF Schurink M Timmer A Kooi EMW Bos AF Intestinal fatty acid-binding protein levels in necrotizing enterocolitis correlate with extent of necrotic bowel: results from a multicenter study. J Pediatr Surg. (2015) 50(7):1115–8. 10.1016/j.jpedsurg.2014.11.037 25783297

[30] Li Z Sheng Z. Significance of dynamic evolution of Tnf-*Α*, Il-6 and intestinal fatty acid-binding protein levels in neonatal necrotizing enterocolitis. Exp Ther Med. (2017) 15(2):1289–92. 29399120 10.3892/etm.2017.5532 PMC5774532

[31] Schurink M Kooi EMW Hulzebos CV Kox RG Groen H Heineman E Intestinal fatty acid-binding protein as a diagnostic marker for complicated and uncomplicated necrotizing enterocolitis: a prospective cohort study. PLoS One. (2015) 10(3). 10.1371/journal.pone.0121336 25793701 PMC4368100

[32] Yang G Wang Y Jiang X. Diagnostic value of intestinal fatty-acid-binding protein in necrotizing enterocolitis: a systematic review and meta-analysis. Indian J Pediatr. (2016) 83(12-13):1410–9. 10.1007/s12098-016-2144-9 27272048

[33] Ng PC. Biomarkers of necrotising enterocolitis. Semin Fetal Neonatal Med. (2014) 19:33–8. 10.1016/j.siny.2013.09.002 24080072

[34] Ng PC. An update on biomarkers of necrotizing enterocolitis. Semin Fetal Neonatal Med. (2018) 23(6):380–6. 10.1016/j.siny.2018.07.006 30082194

[35] Shi L Zhou PH Xi J Yu HG Zhang BH. Recombinant human trefoil factor 3 ameliorates bowel injury: its anti-inflammatory effect on experimental necrotizing enterocolitis. Int J Pept. (2014) 634135. 10.1016/j.jpedsurg.2018.04.034 PMC3944653 24688551

[36] Albanna EA Ahmed HS Awad HA. Stool calprotectin in necrotizing enterocolitis. J Clin Neonatol. (2014) 3(1):16–9. 10.4103/2249-4847.128721 24741535 PMC3982334

[37] Aydemir O Aydemir C Sarikabadayi YU Emre Canpolat F Erdeve O Biyikli Z. Fecal calprotectin levels are increased in infants with necrotizing enterocolitis. J Matern Fetal Neonatal Med. (2012) 25:2237–41. 10.3109/14767058.2012.684172 22524488

[38] Bin-Nun A Booms C Sabag N Mevorach R Algur N Hammerman C. Rapid fecal calprotectin (fc) analysis: point of care testing for diagnosing early necrotizing enterocolitis. Am J Perinatol. (2014) 32(4):337–42. 10.1055/s-0034-1384640 25111039

[39] MacQueen BC Christensen RD Yost CC Lambert DK Baer VL Sheffield MJ Elevated fecal calprotectin levels during necrotizing enterocolitis are associated with activated neutrophils extruding neutrophil extracellular traps. J Perinatol. (2016) 36(10):862–9. 10.1038/jp.2016.105 27388941 PMC5045760

[40] Yoon JM Park JY Ko KO Lim JW Cheon EJ Kim HJ. Fecal calprotectin concentration in neonatal necrotizing enterocolitis. Korean J Pediatr. (2014) 57(8):351–6. 10.3345/kjp.2014.57.8.351 25210522 PMC4155179

[41] Zhang M Zhang X Zhang J. Diagnostic value of fecal calprotectin in preterm infants with necrotizing enterocolitis. Clin Lab. (2016) 62(5):863–9. 10.7754/clin.lab.2015 27349012

[42] Murkin JM Arango M. Near-Infrared spectroscopy as an Index of brain and tissue oxygenation. Br J Anaesth. (2009) 103(Suppl 1):i3–13. 10.1093/bja/aep299 20007987

[43] Banerjee J Aladangady N. Biomarkers to decide red blood cell transfusion in newborn infants. Transfusion. (2014) 54(10):2574–82. 10.1111/trf.12670 24797124

[44] Sandal G Oguz SS Erdeve O Akar M Uras N Dilmen U. Assessment of red blood cell transfusion and transfusion duration on cerebral and mesenteric oxygenation using near-infrared spectroscopy in preterm infants with symptomatic Anemia. Transfusion. (2014) 54(4):1100–5. 10.1111/trf.12359 23901886

[45] Sood BG Cortez J McLaughlin KL Gupta M Amaram A Kolli M Near infrared spectroscopy as a biomarker for necrotizing enterocolitis following red blood cell transfusion. J Near InfraRed Spectrosc. (2014) 22(6):375–88. 10.1255/jnirs.1135

[46] Schaaf JM Liem SM Mol BW Abu-Hanna A Ravelli AC. Ethnic and racial disparities in the risk of preterm birth: a systematic review and meta-analysis. Am J Perinatol. (2013) 30(6):433–50. 10.1055/s-0032-1326988 23059494

[47] Howell EA Janevic T Hebert PL Egorova NN Balbierz A Zeitlin J. Differences in morbidity and mortality rates in black, white, and hispanic very preterm infants among New York City Hospitals. JAMA Pediatr. (2018) 172(3):269–77. 10.1001/jamapediatrics.2017.4402 29297054 PMC5796743

[48] Boghossian NS Geraci M Lorch SA Phibbs CS Edwards EM Horbar JD. Racial and ethnic differences over time in outcomes of infants born less than 30 Weeks’ gestation. Pediatrics. (2019) 144(3):e20191106. 10.1542/peds.2019-1106 31405887 PMC6813804

[49] Janevic T Zeitlin J Auger N Egorova NN Hebert P Balbierz A Association of race/ethnicity with very preterm neonatal morbidities. JAMA Pediatr. (2018) 172(11):1061–9. 10.1001/jamapediatrics.2018.2029 30208467 PMC6248139

[50] Wallace ME Mendola P Kim SS Epps N Chen Z Smarr M Racial/ethnic differences in preterm perinatal outcomes. Am J Obstet Gynecol. (2017) 216(3):306.e1–.e12. 10.1016/j.ajog.2016.11.1026 27865977 PMC5572800

[51] Collins JW David RJ. Racial disparity in low birth weight and infant mortality. Clin Perinatol. (2009) 36(1):63–73. 10.1016/j.clp.2008.09.004 19161865

[52] MacDorman MF Mathews TJ. Understanding racial and ethnic disparities in U.S. Infant mortality rates. NCHS Data Brief. (2011) 74:1–8. 22617114

[53] Huybregts RA de Vroege R Jansen EK van Schijndel AW Christiaans HM van Oeveren W. The association of hemodilution and transfusion of red blood cells with biochemical markers of splanchnic and renal injury during cardiopulmonary bypass. Anesth Analg. (2009) 109(2):331–9. 10.1213/ane.0b013e3181ac52b2 19608799

[54] Van Vonderen JJ Roest AA Siew ML Walther FJ Hooper SB Te Pas AB. Measuring physiological changes during the transition to life after birth. Neonatology. (2014) 105:230–42. 10.1159/000356704 24504011

[55] Gephart SM. Transfusion-Associated necrotizing enterocolitis: evidence and uncertainty. Adv Neonatal Care. (2012) 12(4):232–6. 10.1097/ANC.0b013e31825e20ee 22864004 PMC3414263

[56] European Medicines Agency. Report on the expert meeting on neonatal and paediatric sepsis. (2010). p. 1–6. Available online at: https://www.ema.europa.eu/en/documents/report/report-expert-meeting-neonatal-and-paediatric-sepsis_en.pdf (accessed January 06, 2023).

[57] Bailey SM Hendricks-Munoz KD Wells JT Mally P. Packed red blood cell transfusion increases regional cerebral and splanchnic tissue oxygen saturation in anemic symptomatic preterm infants. Am J Perinatol. (2010) 27(6):445–53. 10.1055/s-0030-1247598 20099219

[58] Christensen RD. Association between red blood cell transfusions and necrotizing enterocolitis. J Pediatr. (2011) 158(3):349–50. 10.1016/j.jpeds.2010.10.030 21146187

[59] Stritzke AI Smyth J Synnes A Lee SK Shah PS. Transfusion-associated necrotising enterocolitis in neonates. Arch Dis Child Fetal Neonatal Ed. (2012) 98(1):F10–4. 10.1136/fetalneonatal-2011-301282 22447991

[60] van Hoften JC Verhagen EA Keating P ter Horst HJ Bos AF. Cerebral tissue oxygen saturation and extraction in preterm infants before and after blood transfusion. Arch Dis Child Fetal Neonatal Ed. (2010) 95(5):F352–8. 10.1136/adc.2009.163592 20466739

[61] Dani C Pratesi S Fontanelli G Barp J Bertini G. Blood transfusions increase cerebral, splanchnic, and renal oxygenation in anemic preterm infants. Transfusion. (2010) 50(6):1220–6. 10.1111/j.1537-2995.2009.02575.x 20113454

[62] Banerjee J Leung TS Aladangady N. Effect of blood transfusion on intestinal blood flow and oxygenation in extremely preterm infants during first week of life. Transfusion. (2016) 56(4):808–15. 10.1111/trf.13434 26643925

[63] Banerjee J Leung TS Aladangady NA. Blood transfusion in preterm infants improves intestinal tissue oxygenation without alteration in blood flow. Vox Sang. (2016) 111(4):399–408. 10.1111/vox.12436 27509230

[64] Ferrari M Mottola L Quaresima V. Principles, techniques and limitations of nirs. Can J Appl Physiol. (2004) 29:464–87. 10.1139/h04-031 15328595

[65] Balegar VK Jayawardhana M Martin AJ de Chazal P Nanan RKH. Hierarchical improvement of regional tissue oxygenation after packed red blood cell transfusion. PLoS One. (2022) 17(7):e0271563. 10.1371/journal.pone.0271563 35857790 PMC9299358

[66] Aktas S Ergenekon E Ozcan E Aksu M Unal S Hirfanoglu IM Effects of blood transfusion on regional tissue oxygenation in preterm newborns are dependent on the degree of anaemia. J Paediatr Child Health. (2019) 55(10):1209–13. 10.1111/jpc.14378 30632233

[67] Thuijls G Derikx JP van Wijck K Zimmermann LJ Degraeuwe PL Mulder TL Non-invasive markers for early diagnosis and determination of the severity of necrotizing enterocolitis. Ann Surg. (2010) 251(6):1174–80. 10.1097/SLA.0b013e3181d778c4 20485148

[68] Kanda T Fujii H Tani T Murakami H Suda T Sakai Y Intestinal fatty acid-binding protein is a useful diagnostic marker for mesenteric infarction in humans. Gastroenterology. (1996) 110(2):339–43. 10.1053/gast.1996.v110.pm8566578 8566578

[69] Vukovic V Haugland HK Nicklee T JMA Hedley DW. Hypoxia-inducible factor -1 aplha is an intrinsic marker for hypoxia in cervical cancer xenografts. American Association for Cancer Research. (2001) 15:7394–8. 11606368

[70] Ng E W Poon T C Lam H S Cheung H M Ma T P Chan K Y Gut-Associated biomarkers L-fabp, I-fabp, and Tff3 and lit score for diagnosis of surgical necrotizing enterocolitis in preterm infants. Ann Surg. (2013) 258(6):1111–8. 10.1097/SLA.0b013e318288ea96 23470582

[71] Arthur C Nalbant D Feldman HA Saeedi BJ Matthews J Robinson BS Anemia induces gut inflammation and injury in an animal model of preterm infants. Transfusion. (2019) 59:1233–45. 10.1111/trf.15254 30897226 PMC6525338

[72] Ludewig S Jarbouh R Ardelt M Mothes H Rauchfuß F Fahrner R Bowel ischemia in icu patients: diagnostic value of I-fabp Depends on the interval to the triggering event. Gastroenterol Res Pract. (2017) 2017:2795176. 10.1155/2017/2795176 28630622 PMC5467337

[73] Dani C Poggi C Gozzini E Leonardi V Sereni A Abbate R Red blood cell transfusions can induce proinflammatory cytokines in preterm infants. Transfusion. (2017) 57:1304–10. 10.1111/trf.14080 28295397

[74] Kim-Shapiro DB Lee J Gladwin MT. Storage lesion: role of red blood cell breakdown. Transfusion. (2011) 51(4):844–51. 10.1111/j.1537-2995.2011.03100.x 21496045 PMC3080238

[75] New HV Berryman J Bolton-Maggs PHB Cantwell C Chalmers EA Davies T Guidelines on transfusion for fetuses, neonates and older children. Br J Haematol. (2016) 175(5):784–828. 10.1111/bjh.14233 27861734

[76] Rouge C Butel MJ Piloquet H Ferraris L Legrand A Vodovar M. Fecal calprotectin excretion in preterm infants during the neonatal period. PLoS One. (2010) 5:e11083. 10.1371/journal.pone.0011083 20552029 PMC2884033

[77] Xu W Zhang Y Zhao W Chen J Maas K Hussain N Trends of fecal calprotectin levels and associations with early life experience in preterm infants. Interdisciplinary Nursing Research. (2022) 1(1):36–42. 10.1097/NR9.0000000000000006 36590866 PMC9766919

[78] Franz AR Engel C Bassler D Rüdiger M Thome UH Maier RF Effects of liberal vs restrictive transfusion thresholds on survival and neurocognitive outcomes in extremely low-birth-weight infants: the ettno randomized clinical trial. JAMA. (2020) 324(6):560–70. 10.1001/jama.2020.10690 32780138 PMC7420159

[79] Kirpalani H Bell EF Hintz SR Tan S Schmidt B Chaudhary AS Higher or lower hemoglobin transfusion thresholds for preterm infants. N Engl J Med. (2020) 383(27):2639–51. 10.1056/NEJMoa2020248 33382931 PMC8487591

[80] Cortez J Gupta M Amaram A Pizzino J Sawhney M Sood BG. Non invasive evaluation of splanchnic tissue oxygenation using near-infrared spectroscopy in preterm neonates. J Matern Fetal Neonatal Med. (2011) 24:574–82. 10.3109/14767058.2010.511335 20828232

[81] McNeill S Gatenby JC McElroy S Engelhardt B. Normal cerebral, renal and abdominal regional oxygen saturations using near-infrared spectroscopy in preterm infants. J Perinatol. (2011) 31(1):51–7. 10.1038/jp.2010.71 20539273 PMC3013378

[82] Costeloe K Hardy P Juszczak E Wilks M Millar MR, Probiotics in Preterm Infants Study Collaborative Group. Bifidobacteriu, Breve bbg-001 in very preterm infants: a randomised controlled phase 3 trial. Lancet. (2016) 387:649–60. 10.1016/S0140-6736(15)01027-2 26628328

[83] Naulaers G Morren G Van Huffel S Casaer P Devliegar H. Cerebral tissue oxygenation Index in very premature infants. Arch Dis Child Fetal Neonatal Ed. (2002) 87:F189–F92. 10.1136/fn.87.3.F189 12390989 PMC1721471

[84] Roche-Labarbe N Fenoglio A Aggarwal A Dehaes M Carp SA Franceschini MA Near-Infrared spectroscopy assessment of cerebral oxygen metabolism in the developing premature brain. J Cereb Blood Flow Metab. (2012) 32(3):481–8. 10.1038/jcbfm.2011.145 22027937 PMC3293111

[85] Takami T Sunohara D Kondo A Mizukaki N Suganami Y Takei Y Changes in cerebral perfusion in extremely lbw infants during the first 72 H after birth. Pediatr Res. (2010) 68(5):435–9. 10.1203/PDR.0b013e3181f2bd4d 20657347

[86] Banerjee J Leung TS Aladangady N. Cerebral blood flow and oximetry response to blood transfusion in relation to chronological age in preterm infants. Early Hum Dev. (2016) 97:1–8. 10.1016/j.earlhumdev.2015.10.017 26619762

[87] Bell EF. Red cell transfusion thresholds for preterm infants: finally some answers. Arch Dis Child Fetal Neonatal Ed. (2022) 107(2):126–30. 10.1136/archdischild-2020-320495 33906941

